# Innovative angiography: a new approach to discover more hepatic vein collaterals in patients with cirrhotic portal hypertension

**DOI:** 10.1186/s12876-023-02792-6

**Published:** 2023-05-10

**Authors:** Bowen Liu, Zhendong Yue, Ting Cui, Hongwei Zhao, Lei Wang, Zhenhua Fan, Yifan Wu, Mingming Meng, Ke Zhang, Li Jiang, Huiguo Ding, Yuening Zhang, Fuquan Liu

**Affiliations:** 1 Department of Interventional Therapy, Beijing Shijitan Hospital, Capital Medical University, No.10 Tieyi Road, Yangfangdian Street, Haidian District, Beijing, 100038 China; 2 Department of Interventional Therapy, Beijing Tongren Hospital, Capital Medical University, Beijing, 100005 China; 3 Department of Gastroenterology, Beijing Shijitan Hospital, Capital Medical University, Beijing, 100038 China; 4 Department of General Surgery, Beijing Ditan Hospital, Capital Medical University, Beijing, 100102 China; 5 Department of Gastroenterology, Beijing YouAn Hospital, Capital Medical University, Beijing, 100069 China

**Keywords:** Portal hypertension, Cirrhosis, Portal venous pressure, Hepatic venous pressure gradient

## Abstract

**Background:**

The hemodynamics of patients with cirrhosis and portal hypertension are complex and variable. We aimed to investigate differences in venous pressures determined by innovative angiography and conventional angiography using balloon occlusion of the hepatic veins in patients with alcoholic cirrhosis and portal hypertension.

**Methods:**

A total of 134 patients with alcoholic cirrhosis who fulfilled the inclusion criteria from June 2017 to June 2020 were included. During transjugular intrahepatic portosystemic shunt, conventional and innovative angiography were performed, and venous pressures were measured. A paired *t*-test and Pearson’s correlation coefficient were used for analysis.

**Results:**

Conventional and innovative hepatic angiography detected lateral branches of the hepatic vein in 26 (19.4%) and 65 (48.5%) cases, respectively (*P* < 0.001). Innovative angiography detected a total of 65 patients with lateral shunts, of whom 37 (56.9%) had initial shunts. The average wedged hepatic venous pressure and portal venous pressure of the initial lateral branches were 21.27 ± 6.66 and 35.84 ± 7.86 mmHg, respectively, with correlation and determination coefficients of 0.342 (*P* < 0.05) and 0.117, respectively. The mean hepatic venous pressure gradient and portal pressure gradient were 9.59 ± 7.64 and 26.86 ± 6.78 mmHg, respectively, with correlation and determination coefficients of 0.292 (*P* = 0.079) and 0.085, respectively.

**Conclusions:**

Innovative angiography reveals collateral branches of the hepatic veins more effectively than conventional angiography. Hepatic vein collateral branches are the primary factors leading to underestimation of wedged hepatic venous pressures and hepatic venous pressure gradients, with the initial hepatic vein collateral branches resulting in the most severe underestimations.

## Background

Hepatitis and alcoholism are common causes of cirrhotic portal hypertension [1, 2]. Hepatocytes are damaged by prolonged heavy drinking, and a series of pathological changes in the liver may occur, resulting in increased portal vein pressure and, eventually, leading to a series of clinical symptoms, such as esophageal gastric varices, gastrointestinal bleeding, and ascites [3, 4]. These symptoms are directly related to a gradual increase in portal vein pressure. The most accurate diagnosis and prognosis prediction for patients should be the direct measurement of portal vein pressure. However, precise measurement of portal vein pressure is complicated, traumatic, and technically demanding; therefore, it is difficult to routinely apply in clinical practice.

In recent years, the measurement of hepatic vein pressure in lieu of portal vein pressure has been used clinically and in research. The hepatic venous pressure gradient (HVPG) can be used as the "gold standard" to indirectly reflect the portal pressure gradient (PPG) [4–7]. Nevertheless, few studies have been conducted on the correlation between the HVPG and PPG in clinical practice. It has been suggested that there is a certain correlation between wedged hepatic venous pressure (WHVP) and portal venous pressure (PVP) in patients with portal hypertension and alcoholic cirrhosis [8], but these cases are limited. There is a standard for measuring hepatic venous pressure; injection of contrast agent (5 mL) after balloon occlusion of the hepatic vein is an important component of the standardized procedure for observing hepatic vein collateral branches and hepatic vein occlusion. It has been reported that the presence of collateral branches of the hepatic vein affects the accuracy of WHVP, but the detection rate is relatively low [9].

In this study, 134 patients with alcoholic cirrhosis and portal hypertension who fulfilled the inclusion criteria from June 2017 to June 2020 were enrolled for transjugular intrahepatic portosystemic shunt (TIPS) and underwent innovative hepatic venous angiography based on conventional measurement of hepatic venous pressure and angiography. Subsequently, we analyzed the correlation between the hepatic vein anatomy and venous pressure.

## Methods

### Patients

A total of 134 (males: 119 [88.8%], females: 15 [11.2%]; mean age: 55.02 ± 10.65 [range: 19–75] years) patients with alcoholic cirrhosis who fulfilled the inclusion criteria for portal hypertension underwent TIPS between January 2017 and June 2020. PPG and HVPG were obtained by measuring various pressures during the interventional surgery. There were 88 cases of gastrointestinal hemorrhage in patients with alcoholic cirrhosis and portal hypertension (65.7%), 35 cases of intractable ascites or pleural effusion combined with ascites (26.1%), and 11 cases of gastrointestinal bleeding with intractable ascites (8.2%). According to Child–Pugh classification, there were 36 grade A, 47 grade B, and 51 grade C cases. Fourteen cases of alcoholic cirrhosis with portal hypertension were complicated with liver cancer. The study protocol conforms to the ethical guidelines of the 1975 Declaration of Helsinki (6th revision, 2008) and was approved by the Ethics Committee of Beijing Shijitan Hospital (2018(01)). Informed consent was obtained from each patient included in the study.

### Inclusion criteria

The inclusion criteria were as follows: 1) TIPS indications, 2) age 18–75 years, 3) presence of TIPS and undergoing elective surgery, and 4) normal hepatic vein and inferior vena cava.

### Exclusion criteria

The exclusion criteria were as follows**:** 1) portal vein carcinoma thrombus, 2) arterioportal venous fistula, 3) portal vein thrombosis affecting blood flow (generally exceeding 1/3 of the portal vein), 4) use of drugs affecting portal vein pressure within the preceding week, and 5) presence of factors that affect the accuracy of intraoperative pressure measurement, such as biliary-cardiac reflex and incomplete balloon occlusion.

### Method of pressure measurement

#### Preoperative preparation

Various examinations were conducted before the surgery, including routine blood examination, hepatorenal function, ICG-R15 (quantitative determination of liver function, retention rate of indocyanine green in 15 min), blood ammonia, blood type, electrocardiograph, coagulation function, portal vein ultrasound, and abdominal CT and/or magnetic resonance imaging. Coagulation function, platelet count, bilirubin, albumin, and hemoglobin levels should be adjusted according to the interventional surgery. The effects and risks of the surgery were explained to patients and their families, and consent for surgery was obtained. Medications affecting portal vein pressure were discontinued for at least one week preceding surgery.

#### Pressure measurement

##### Conventional methods of measuring pressure [10, 11]

Pressure was measured by catheterization of the right internal jugular vein to the right atrium, inferior vena cava, and hepatic vein under routine disinfection and local anesthesia. A Fogarty balloon catheter (Edward Company, USA) was inserted into the hepatic vein using an RUPS-100 outer sheath (COOK Company, USA). The tip of the balloon catheter was placed 3–5 cm from the hepatic vein to the opening of the inferior vena cava. WHVP and FHVP were measured before and after hepatic vein occlusion by expanding the balloon (contrast agent (5 mL) was carefully injected). The pressure was recorded after stabilization; the pressure measurement was repeated three times, and the average value was recorded; the HVPG value was then calculated. The liver parenchyma and portal vein were punctured through the hepatic vein or inferior vena cava. After successful portal vein puncture, a porcine tail catheter was inserted into the splenic or superior mesenteric vein for angiography. Before the shunt, the main portal vein pressure was measured three times; the average value was recorded and the PPG value was then calculated.

##### Innovative method of angiography

After completion of the standard measurement methods, the hepatic vein was blocked again by inserting a balloon at the same position as was done in the conventional method. The dose of contrast agent was increased (5 mL/s up to a total of 15 mL), and pressure angiography was performed; the pressure was 200–300 psi, and continuous angiography was performed for more than 6 s. Subsequently, subtraction was performed. The WHVP and free hepatic venous pressure (FHVP) were measured again; the pressure was recorded after stabilization, and the measurement was repeated three times; the average value was used to calculate the HVPG value.

##### Precautions

To observe the occlusion of the balloon catheter after balloon dilatation, if the blockage was incomplete, the position was adjusted and retested; radiography of the balloon catheter was performed. The indwelling catheter in the portal vein was inserted for 24–48 h; the portal vein pressure was measured at least three times per day. The pressures of the inferior vena cava and right atrium were measured three times during extubation, and the average value was recorded.

### Statistical analysis

GraphPad Prism 8 (GraphPad, Inc., La Jolla, CA, USA) was used for the statistical analysis. The paired *t*-test was used to analyze the differences between WHVP and PVP, PPG and HVPG, and FHVP and IVCP. The Pearson correlation test was applied to analyze the correlation and determination coefficients. Blood pressures are expressed as means ± standard deviations. *P* < 0.05 was considered statistically significant.

## Results

### Differences between conventional and innovative angiography

**Fig. 1:**
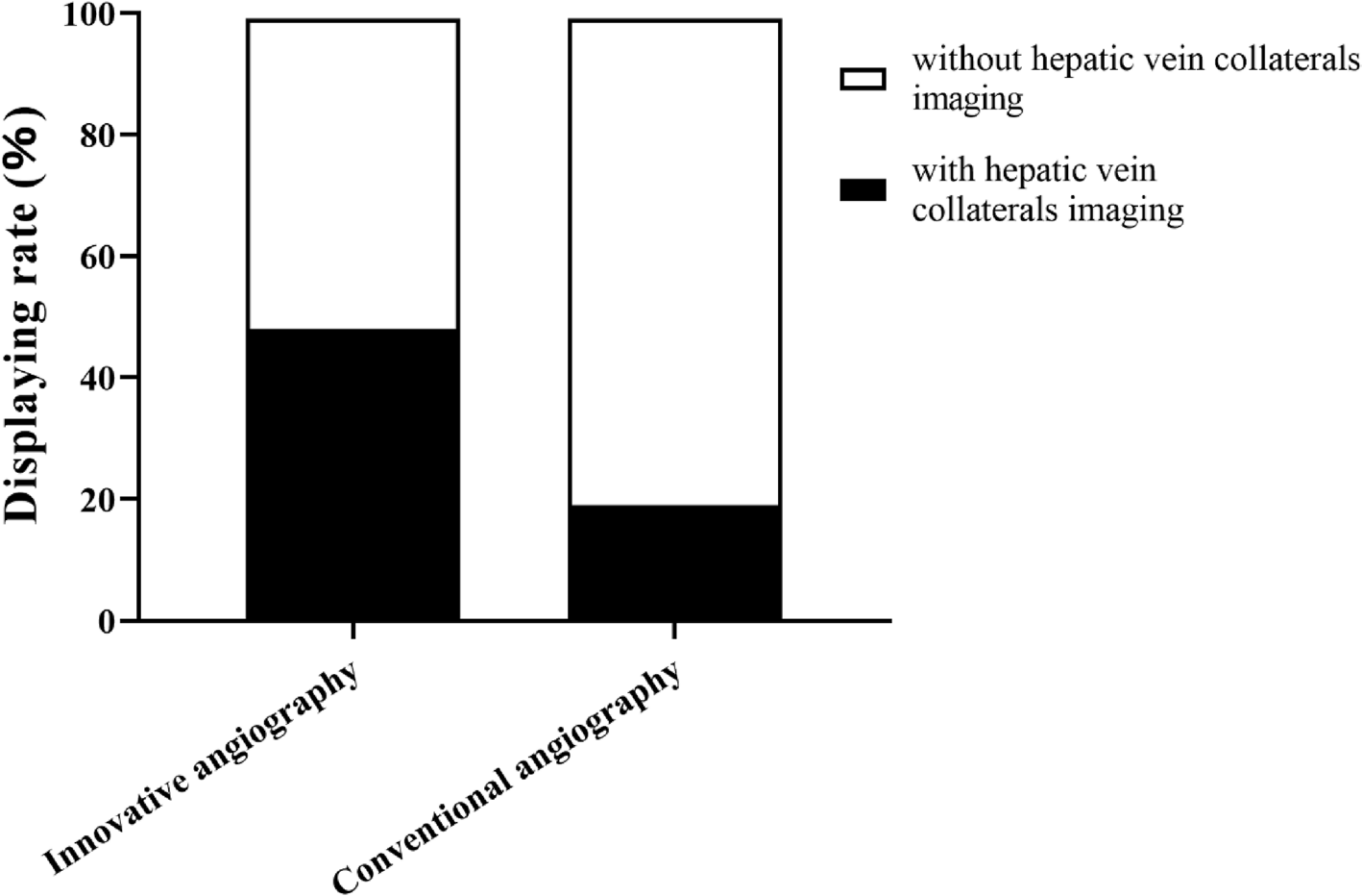
Comparison of hepatic vein collateral branches in innovative and conventional angiography (*P* < 0.001)

Conventional and innovative hepatic angiography detected hepatic vein collateral branches in 26 (19.4%) and 65 (48.5%) cases, respectively. The difference was statistically significant (*P* < 0.001) (Fig. 1).

### Individual pressure correlations in patients with collateral vessels detected by conventional angiography

**Fig. 2:**
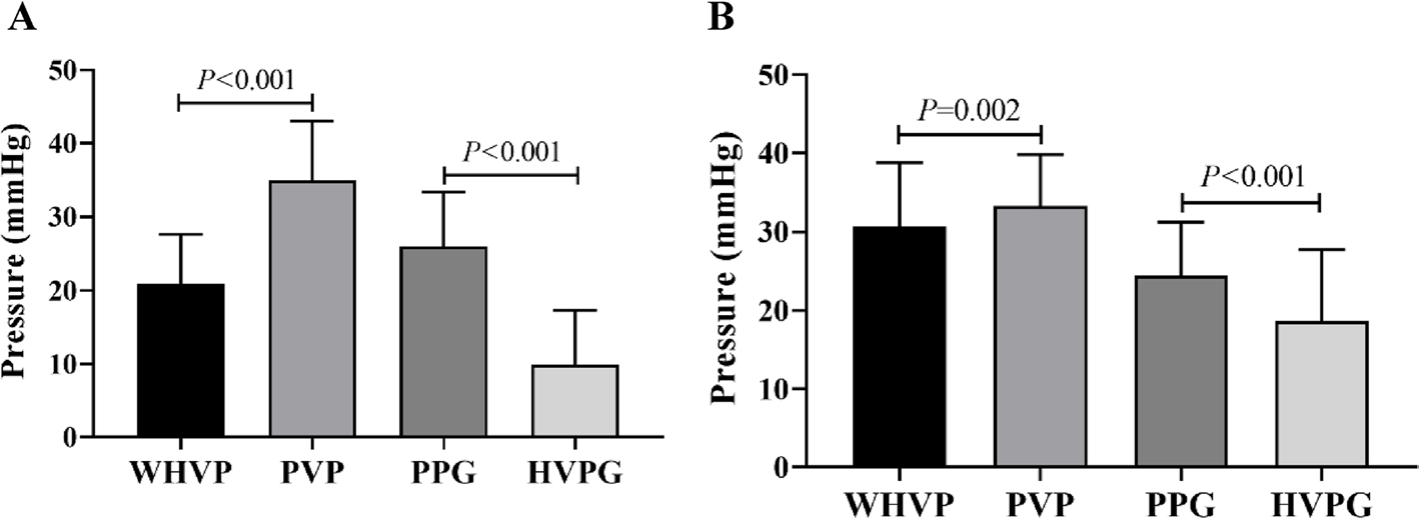
Pressure relationships between venous pressure in patients undergoing conventional angiography. **A** With lateral hepatic vein development and (**B**) Without lateral hepatic vein development (WHVP, wedged hepatic venous pressure; PVP, portal venous pressure; HVPG, hepatic venous pressure gradient; PPG, portal pressure gradient)

**Fig. 3:**
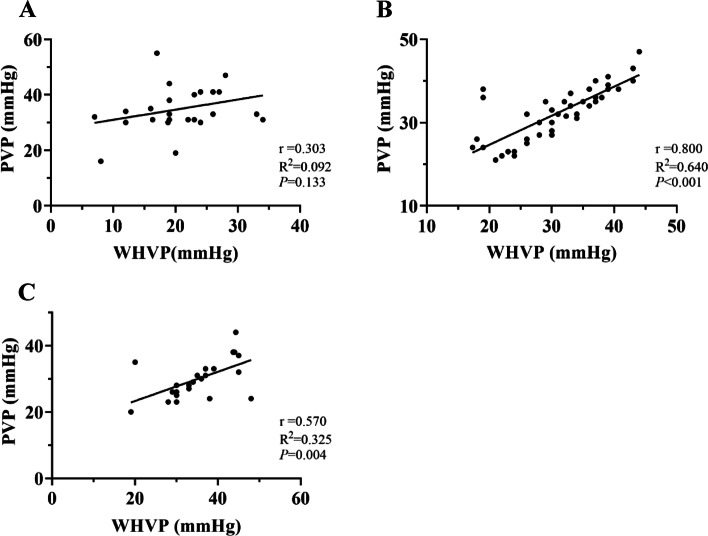
Correlation between WHVP and PVP under different collateral development methods. **A** patients with collateral vessels detected by conventional angiography, (**B**) patients with portal vein visualization using innovative angiography, and (**C**) patients without visualization using innovative angiography (WHVP, wedged hepatic venous pressure; PVP, portal venous pressure; HVPG, hepatic venous pressure gradient; PPG, portal pressure gradient)

**Fig. 4:**
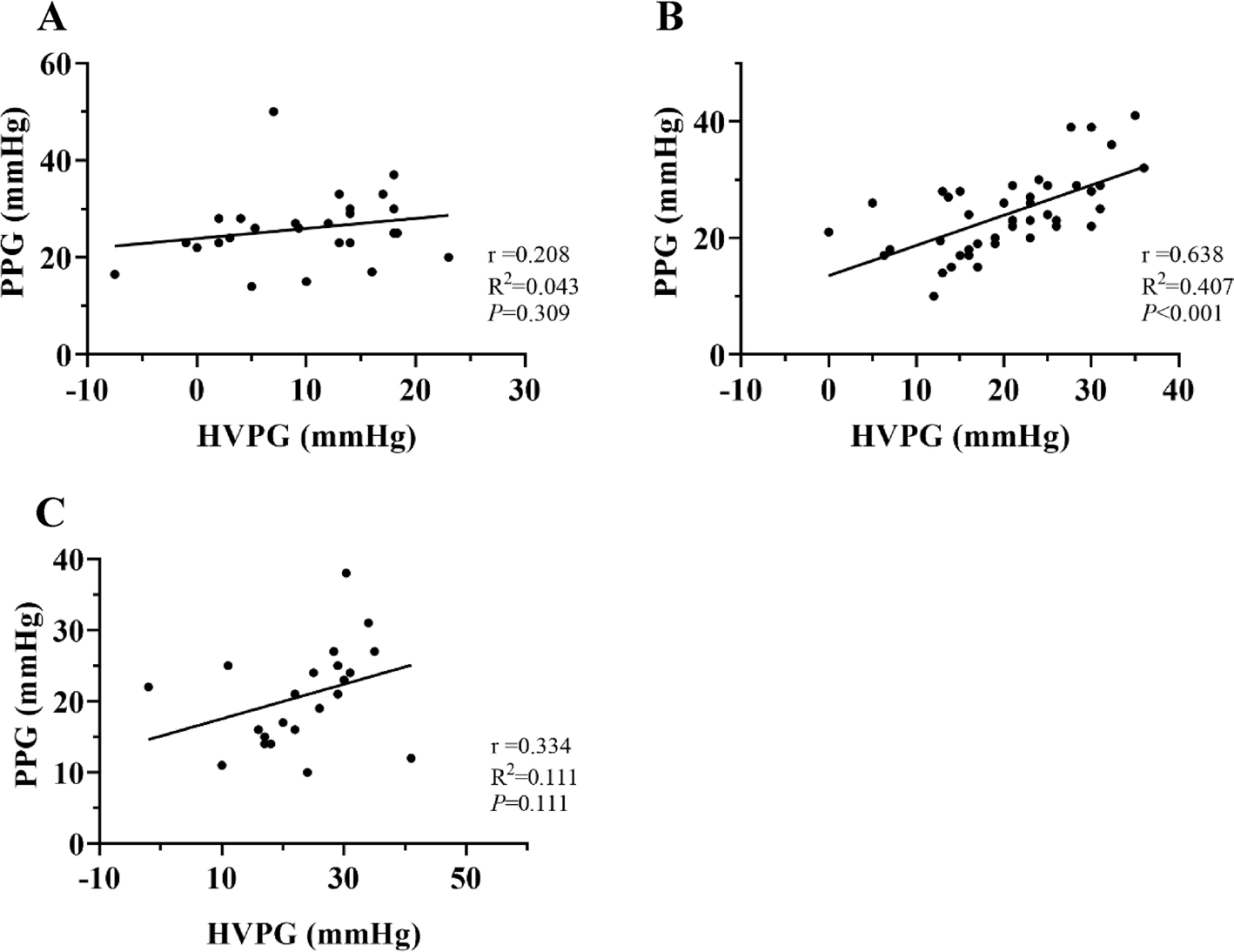
Correlation between HVPG and PPG under different collateral development methods. **A** patients with collateral vessels detected by conventional angiography, (**B**) patients with portal vein visualization using innovative angiography, and (**C**) patients without visualization using innovative angiography (WHVP, wedged hepatic venous pressure; PVP, portal venous pressure; HVPG, hepatic venous pressure gradient; PPG, portal pressure gradient)

Twenty-six patients with collateral branches underwent conventional angiography (Fig. 2A). The average WHVP and PVP were 20.89 ± 6.69 and 34.96 ± 8.08 mmHg (Table 1); the correlation and determination coefficients were 0.303 (*P* = 0.133) and 0.092, respectively (Fig. 3A). The average HVPG and PPG were 9.86 ± 7.44 and (25.94 ± 7.42) mmHg, respectively (Table 2). The coefficients of correlation and determination were 0.208 (*P* = 0.309) and 0.043, respectively (Fig. 4A).

**Table 1:** Correlation between WHVP and PVP in lateral branch imaging of different hepatic veins

	WHVP (mmHg)	PVP (mmHg)	r	R^2^	*P*-value
Conventional angiography	20.89 ± 6.69	34.96 ± 8.08	0.303	0.092	0.133
Early shunting	21.27 ± 6.66	35.84 ± 7.86	0.342	0.117	0.038
Middle shunting	28.79 ± 6.94	35.64 ± 4.51	0.208	0.043	0.353
Late shunting	30.50 ± 8.31	34.83 ± 8.11	0.995	0.991	< 0.001
Portal vein development	31.07 ± 7.53	32.39 ± 6.58	0.800	0.640	< 0.001
No shunting	35.49 ± 7.78	30.13 ± 6.01	0.570	0.325	0.004

**Table 2:** Correlation between HVPG and PPG in lateral branch imaging of different hepatic veins

	HVPG (mmHg)	PPG (mmHg)	r	R^2^	*P*-value
Conventional angiography	9.86 ± 7.44	25.94 ± 7.42	0.208	0.043	0.309
Early shunting	9.59 ± 7.64	26.86 ± 6.78	0.292	0.085	0.079
Middle shunting	15.62 ± 5.39	26.23 ± 5.70	0.264	0.069	0.236
Late shunting	17.00 ± 10.02	27.33 ± 7.97	0.779	0.607	0.068
Portal vein development	20.18 ± 8.31	23.99 ± 6.75	0.638	0.407	< 0.001
No shunting	23.50 ± 9.30	20.83 ± 6.78	0.334	0.111	0.111

### Initial shunt: the most significant factor causing underestimation of WHVP and HVPG in innovative angiography

**Fig. 5:**
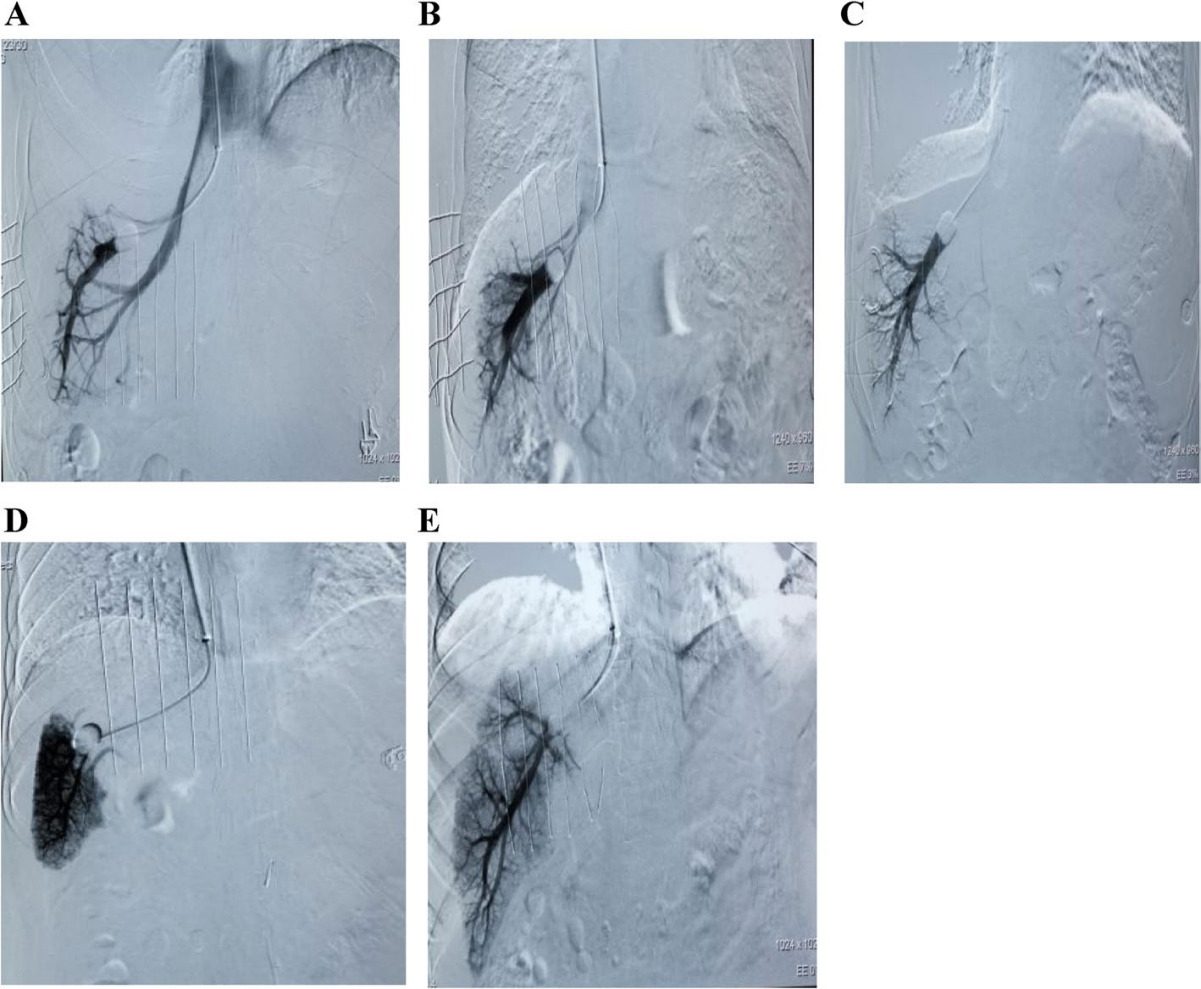
Five different development states under innovative angiography. **A** Initial hepatic vein collateral formation, (**B**) Mid-term hepatic vein collateral formation, (**C**) Late hepatic vein collateral formation, (**D**) Portal vein imaging, and (**E**) Absence of hepatic vein collateral branches. *Note: the hepatic vein in innovative angiography: (1) initial shunt: collateral visualization was seen within 2 s after beginning angiography; (2) mid-term shunt: collateral visualization was seen 3–4 s after initiating angiography; (3) late shunt: collateral visualization was seen 5–6 s after beginning angiography; (4) visualization of the portal vein: visualization of the portal vein in the process of angiography; (5) no collateral branches: no collateral visualization in the process of angiography

**Fig. 6:**
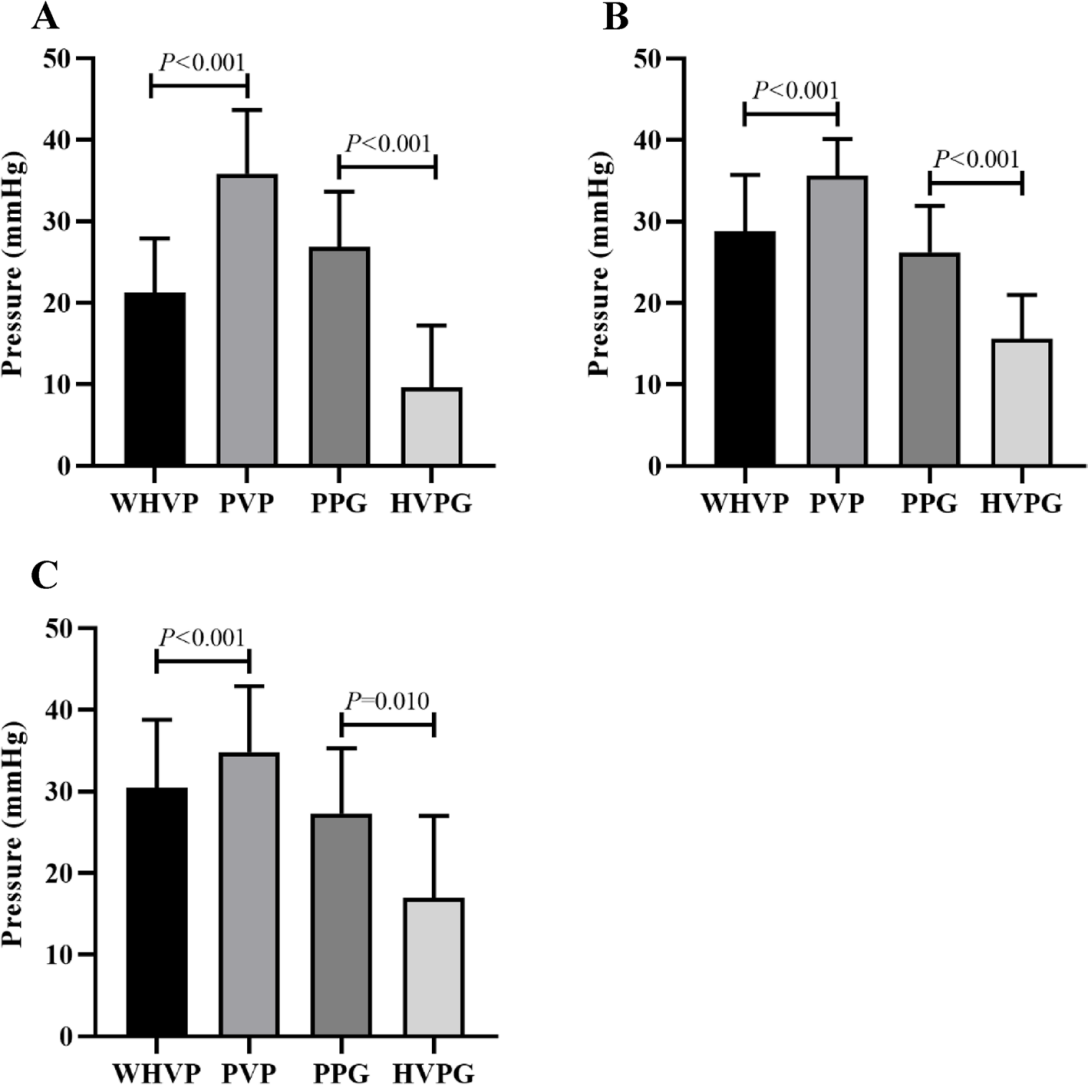
Pressure relationships in 65 patients with lateral branches detected using innovative angiography. **A** Initial shunt, (**B**) Mid-term shunt, and (**C**) Late shunt (WHVP, wedged hepatic venous pressure; PVP, portal venous pressure; HVPG, hepatic venous pressure gradient; PPG, portal pressure gradient)

Among the 65 patients with lateral branches detected by innovative angiography, 37 cases (56.9%) (Fig. 5A) possessed initial shunts, 22 cases (33.8%) (Fig. 5B) had mid-term shunts, and 6 cases (9.2%) (Fig. 5C) exhibited late shunts (Fig. 6). For the initial shunt, average WHVP and PVP were 21.27 ± 6.66 and 35.84 ± 7.86 mmHg, respectively, with determination coefficients of 0.342 (*P* = 0.038) and 0.117, respectively (Table 1). The HVPG and PPG were 9.59 ± 7.64 and 26.86 ± 6.78 mmHg, respectively, with correlation and determination coefficients of 0.292 (*P* = 0.079) and 0.085, respectively (Table 2). For the middle shunts, the mean WHVP and PVP were 28.79 ± 6.94 and 35.64 ± 4.51 mmHg, respectively, with correlation and determination coefficients of 0.208 (*P* = 0.353) and 0.043, respectively (Table 1). The average HVPG and PPG were 15.62 ± 5.39 and 26.23 ± 5.70 mmHg, respectively, with correlation and determination coefficients of 0.264 (*P* = 0.236) and 0.069, respectively (Table 2). The mean WHVP and PVP of hepatic collaterals in cases with late shunts were 30.50 ± 8.31 and 34.83 ± 8.11 mmHg, respectively, with correlation and determination coefficients of 0.995 (*P* < 0.001) and 0.991, respectively (Table 1). The average HVPG and PPG were 17.00 ± 10.02 and 27.33 ± 7.97 mmHg, respectively with correlation and determination coefficients of 0.779 (*P* = 0.068) and 0.607, respectively (Table 2).

### Venous pressure without collaterals on conventional angiography

There were 108 patients (81.6%) without collateral branches on conventional hepatic venography (Fig. 2B). The average WHVP and PVP were 30.65 ± 8.17 and 33.25 ± 6.60 mmHg, respectively, with correlation and determination coefficients of 0.368 (*P* < 0.001) and 0.135, respectively. The mean HVPG and PPG were 18.67 ± 9.05 and 24.44 ± 6.79 mmHg, respectively, with correlation and determination coefficients of 0.263 (*P* = 0.006) and 0.069, respectively.

### Innovative angiography for various venous pressure situations without collateral vessels

**Fig. 7:**
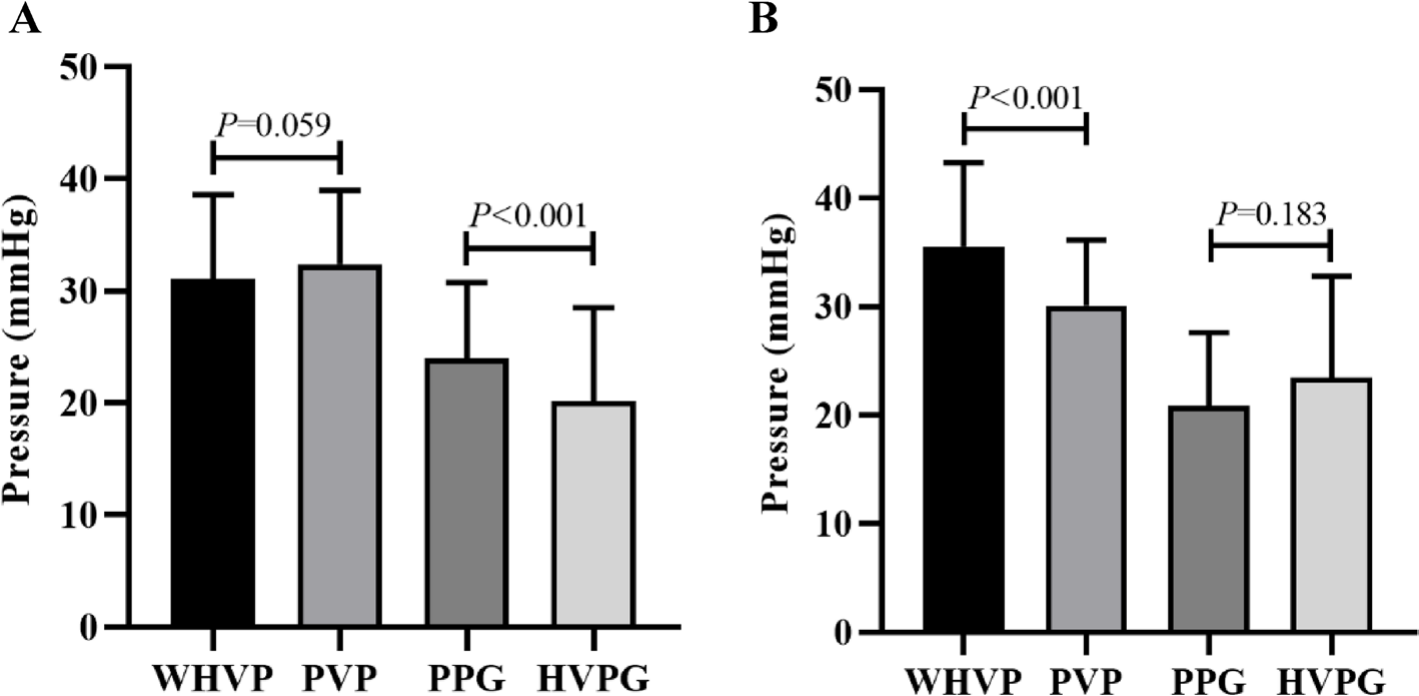
Pressure relationships in 69 patients without collateral branches on innovative hepatic angiography. **A** With portal vein imaging and (**B**) Without portal vein imaging (WHVP, wedged hepatic venous pressure; PVP, portal venous pressure; HVPG, hepatic venous pressure gradient; PPG, portal pressure gradient)

Sixty-nine patients (51.5%) underwent innovative hepatic venography without detection of collaterals, including 45 patients (65.2%) (Fig. 7A) with portal vein visualization (Fig. 5D) and 24 patients (34.8%) (Fig. 7B) without visualization (Fig. 5E). The mean WHVP and PVP of the 45 patients with portal vein imaging were 31.07 ± 7.53 and 32.39 ± 6.58 mmHg (Table 1), respectively, with correlation and determination coefficients of 0.800 (*P* < 0.001) and 0.640, respectively (Fig. 3B). The average HVPG and PPG were 20.18 ± 8.31 and 23.99 ± 6.75 mmHg (Table 2), with correlation and determination coefficients of 0.638 (*P* < 0.001) and 0.407, respectively (Fig. 4B). The mean WHVP and PVP of the 24 patients without portal vein visualization were 35.49 ± 7.78 mmHg and 30.13 ± 6.01 mmHg (Table 1), respectively, with correlation and determination coefficients of 0.570 (*P* = 0.004) and 0.325, respectively (Fig. 3C). The average HVPG and PPG were 23.50 ± 9.30 and 20.83 ± 6.78 mmHg (Table 2), respectively, with correlation and determination coefficients of 0.334 (*P* = 0.111) and 0.111, respectively (Fig. 4C).

## Discussion

Alcoholic cirrhosis is a common cause of portal hypertension. Hemodynamic changes in portal hypertension caused by pathological changes in alcoholic cirrhosis occur mainly in the hepatic sinus, resulting in portal vein resistance and increased blood volume [12, 13]. Normal liver hemodynamics are that the PVP is higher than or equal to the hepatic sinusoidal pressure, WHVP is equal to the hepatic sinusoidal pressure, and the FHVP is higher than the IVCP by 0.5–1.0 mmHg [14–16]. PPG is more meaningful and accurate than PVP in predicting the risk of complications associated with portal hypertension [17]. Theoretically, the HVPG can accurately reflect the PPG. It is preferred to represent PPG because it is simple, is associated with minimal trauma, and is easily accepted by patients [4, 18, 19]. There are significant changes in the structure of the liver tissue, hepatic lobules, and microvessels in patients with alcoholic cirrhosis and portal hypertension [2, 20, 21]. In pathological conditions and clinical practice, whether WHVP accurately represents PVP remains controversial in reports [22].

However, it has rarely been reported whether the HVPG represents PPG. The basic principle of measuring WHVP is that after the hepatic vein is completely blocked by a balloon, hepatic vein pressure gradually increases until it achieves the pressure of the hepatic sinuses; this accurately represents the pressure of the hepatic sinuses [22, 23]. However, in the process of measurement, certain objective and subjective factors can impede accurate measurement. For example, in the presence of collateral branches of the hepatic vein, the blood does not achieve the pressure of the hepatic sinusoid, but blood flows from the collateral branches to other hepatic veins or accessory hepatic veins into the inferior vena cava, or may even flow away quickly in the preliminary stage [16, 24]. In addition, among the subjective factors, if balloon sealing is insufficient to enable the pressure of the hepatic sinusoid to be reached and the front end of the balloon catheter is not free in the blood vessel, the accuracy of the measured pressure is directly affected. Based on the principle of measuring WHVP, as long as lateral branches of the hepatic vein exist, it is possible that the WHVP will not truly represent the pressure of the hepatic sinus.

By using innovative hepatic venography in this study, we detected more collateral branches of the hepatic vein (48.5%) than those identified using the conventional method (19.4%). Furthermore, in patients with collateral branches of the hepatic vein, the WHVP was significantly lower than the PVP and HVPG was significantly lower than PPG. Hepatic vein collateral flow is the key factor causing underestimation of WHVP and HVPG. Moreover, our analysis revealed that hepatic vein collateral branches appeared at the three shunt levels, and this underestimation was more obvious the earlier the shunt appeared. In addition to identifying more cases with hepatic vein collaterals, 45 patients (33.6% of total cases) underwent portal vein imaging in the innovative hepatic venography group in this study. The correlation between WHVP and PVP in these patients was good, and the same was true for the HVPG and PPG. This was because the pressure of the hepatic vein increased after it was filled with the contrast agent. When the pressure of the hepatic vein was balanced with that of the hepatic sinus, the contrast agent entered the portal vein through the hepatic sinus countercurrent, which enabled portal vein imaging, indicating that the hepatic sinus pressure was roughly equal to the portal vein pressure. Furthermore, 24 patients (17.9% of the total cases) had no hepatic vein collateral branches. The mean WHVP of these patients (35.49 ± 7.78 mmHg) was higher than the mean PVP (30.13 ± 6.01 mmHg), with correlation and determination coefficients of 0.570 (*P* = 0.004) and 0.325, respectively. The mean HVPG (23.50 ± 9.30 mmHg) was higher than the PPG (20.83 ± 6.78 mmHg); the correlation and determination coefficients were 0.334 (*P* = 0.111), and 0.111, respectively. Since the hepatic vein was perfused with contrast medium, the hepatic venous pressure gradually increased, and the contrast medium could not enter the portal vein through the hepatic sinusoids (possibly the blood of the hepatic sinusoids is mainly supplied by the hepatic artery) because of the higher hepatic sinusoidal pressure. The contrast medium was retained in the hepatic vein and could not enter the portal vein or the hepatic venous collateral return. Therefore, the absence of collateral branches of the hepatic vein is an important reason for the overestimation of WHVP and HVPG. Studies [9, 25, 26] have reported that patients with WHVP higher than PVP have adverse hepatic blood flow, umbilical vein opening, portocaval anastomotic branches, and gastro-renal shunts. Among the patients in this study, only two had this condition.

Regarding the measurement of free pressure in the inferior vena cava, hepatic vein, and portal vein, the factors affecting the accuracy of measurement are mainly subjective [19, 25, 27]. For example, preoperative health education should be incorporated so that patients can be fully prepared psychologically. Patients who choose an elective operation should be prepared, and patients with massive ascites should ensure drainage of the ascites appropriately and discontinue drugs that affect venous pressure (non-selective beta-blockers influence PVP [28–30], deep sedation using propofol impacts the PPG in patients [31, 32]). During the surgery, the accuracy of the pressure measurement was also affected by the use of local anesthesia and the consistent position of the catheter for multiple pressure measurements. Therefore, it is imperative to pay special attention to patients with the biliary-cardiac reflex and incomplete balloon occlusions. These patients should be excluded if these conditions cannot be corrected.

## Conclusions

In conclusion, innovative hepatic vein angiography can identify more cases of hepatic vein collateral branches in alcoholic cirrhosis with portal hypertension than conventional angiography. Hepatic vein collateral branches are the key factors leading to underestimation of WHVP and HVPG, of which the initial hepatic vein collateral branches exhibit the most profound impact, followed by the middle and late hepatic vein collateral branches. Additionally, this innovative method demonstrated good correlation of WHVP with PVP and HVPG with PPG in patients with portal vein visualization. The absence of hepatic vein collaterals is an important factor resulting in the overestimation of the WHVP and HVPG. However, whether additional factors exist requires further investigation. The total amount of contrast agent needed, injection dose (per second), and injection pressure that are most appropriate for innovative angiography also deserve further investigation.

## Data Availability

The data that support the findings of this study are available from the corresponding author, upon reasonable request.

## Abbreviations

HVPG: Hepatic venous pressure gradient
PPG: Portal pressure gradient
WHVP: Wedged hepatic venous pressure
PVP: Portal venous pressure
TIPS: Transjugular intrahepatic portosystemic shunt
FHVP: Free hepatic venous pressure
IVCP: Inferior vena cava pressure
